# Endodontic Management of a Maxillary First Molar with Two Palatal Canals and a Single Buccal Canal: A Case Report

**DOI:** 10.1155/2012/389387

**Published:** 2012-12-12

**Authors:** Leila Atash biz Yeganeh, Mamak Adel, Reza Vahedi, Maryam Tofangchiha

**Affiliations:** Department of Endodontics, School of Dentistry, Qazvin University of Medical Sciences, Qazvin, Iran

## Abstract

Thorough knowledge of root canal morphology is essential for the endodontic therapy. There are rare variations in canal number and configuration in maxillary molars, which could affect treatment outcome. This paper presents the endodontic management of a maxillary first molar with two palatal canals in one root (Vertucci type IV) and a single buccal canal. In this paper cone-beam computed tomography was made to asses this morphology. This paper is intended to reinforce clinician's awareness of the rare morphology of root canals.

## 1. Introduction

A thorough knowledge of root canal morphology is an important aspect of root canal treatment. Failures to detect aberrant root canals and to identify extra roots are one of the main causes for failure in the endodontic therapy [1]. Human molars demonstrate relatively high anatomic variations and abnormalities with respect to number of roots and root canals. The literature delineates wide variations in root canal morphology of maxillary first molars. Cleghorn et al. (2006) conducted a comprehensive review of the root and root canal morphology of the human permanent maxillary first molar [2]. Limited number of cases with 2 palatal roots/canals (Table 1) and cases with fused buccal roots have been reported [2, 3]. The frequency of a maxillary first molar with two roots or two palatal canals is very low, 3.9 and 1% respectively [2]. A brief review of recent case reports of extra palatal canals in maxillary first molars is presented in Table 1.

Baratto-Filho et al. (2009) assessed internal morphology of maxillary first molars by 3 different methods [4]. They found that second palatal canal prevalence in *ex vivo* assessment, 2.05%, in clinical assessment, 0.65%, and by cone-beam computed tomography, 4.55%.

Some of the previous case reports were based on the radiographic examinations of the teeth. Since the radiographic image is a shadow and it is a 2-dimensional image, the best applicative method for precise clarification of the root canal morphology of a tooth is cone-beam computed tomography (CBCT) [5], however it may not to be practical in all clinical situations. This technique offers significant advances in anatomical reconstruction of teeth before and after instrumentation and obturation.

This case report describes nonsurgical therapy of an unusual permanent maxillary first molar with 3 canals: 2 separate palatal canals and one buccal canal, a morphology that has not been reported in published articles that we found in our search. The root canal anatomy was confirmed with the help of spiral CT.

## 2. Case Report

**Figure 1:**
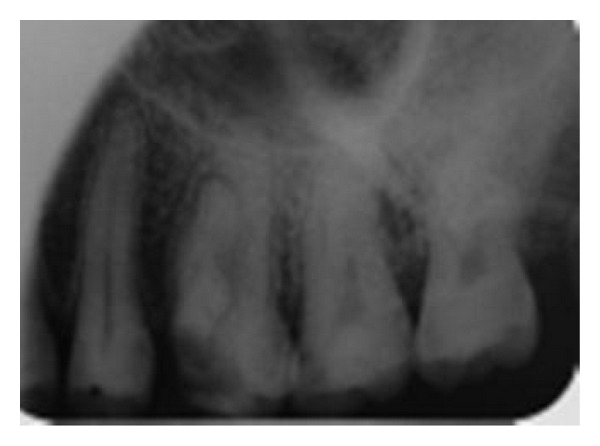
Preoperative radiograph.

A 36-year-old male patient referred to the Department of Endodontics of Qazvin University of Dentistry, Iran for implementation of root canal treatment of right maxillary first molar (tooth no. 3), due to carious exposure of the pulp, with a chief complaint of “cavity in the maxillary right first molar but without any history of pain.” His medical history was not contributory. Clinically the tooth had a deep carious lesion on the mesiocclusal surface. The tooth was not tender to percussion. There was no mobility and periodontal probing was within normal limits. Pulp vitality testing of the involved teeth with cold (DENRONIC, Aeronova GmbH & Co. KG, Germany) and electric pulp stimulation (Parkel Electronics Division, Farmingdale, NY, USA) were negative, indicative of pulp necrosis. The initial periapical radiograph revealed widening of the apical periodontium and was indicative of an unusual complex root canal anatomy (Figure 1). These findings led to a provisional diagnosis of pulp necrosis with chronic apical periodontitis of the right maxillary first molar, necessitating endodontic therapy.

The patient received local anesthesia of 2% lidocaine with 1 : 80000 epinephrine (Persocaine-E, Darou Pakhsh, Iran). After removing caries of the tooth, a conventional endodontic access opening was made. Rubber dam was placed. Clinical examination with a DG-16 endodontic explorer (Hu-Friedy, Chicago, IL, USA) revealed 3 distinct orifices: two palatals with one large buccal orifice. The conventional triangular access was modified to a trapezoidal shape to improve access to the palatal canals and extended to explore the likely second buccal canal. Surprisingly, clinical and radiographic evaluations confirmed the presence of only one buccal root canal. 

**Figure 2:**
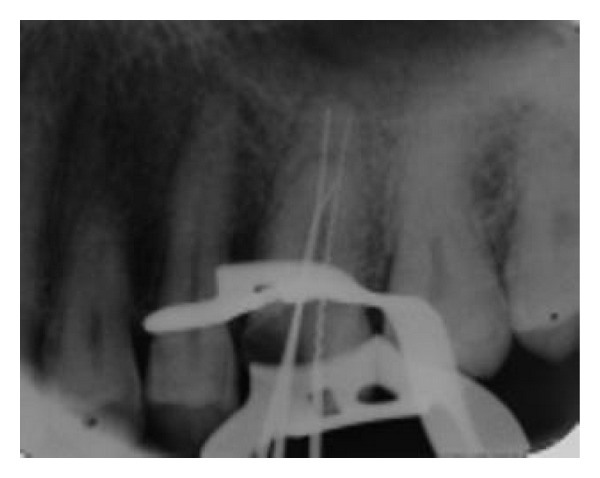
Length-determination radiograph.

**Figure 3:**
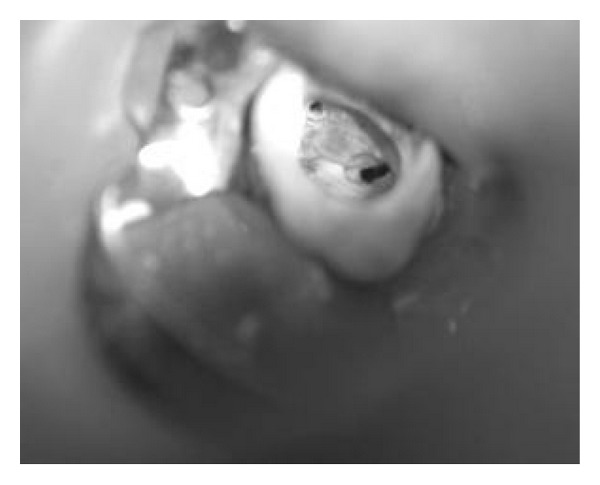
Pulp chamber floor after root canal preparation.

**Figure 4:**
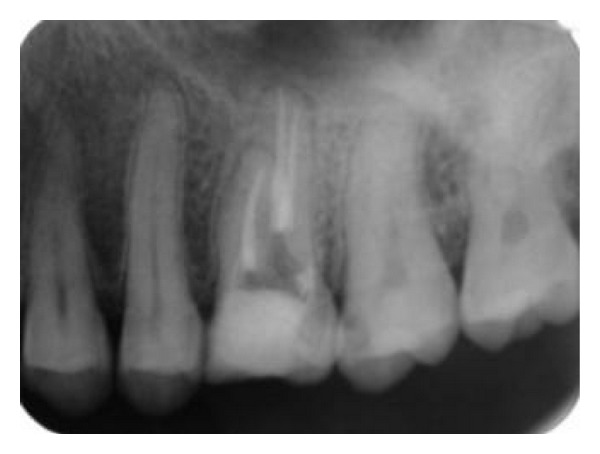
Postoperative radiograph.

After scouting the canals with no.10 and no.15 K-files (Mani INC, Tochigi, Japan), coronal flaring with Protaper Universal Shaping file Sx and S1 (Dentsply, Maillefer, Switzerland) was done. Working lengths were estimated by means of an apex locator (Root ZX, J. Morita Mfg Corp, Kyoto, Japan) and confirmed with a perapical radiograph (Figure 2). The canals were initially instrumented to a size no.15 K-file (Mani INC, Tochigi, Japan), under copious irrigation with 2.5% sodium hypochlorite. Biomechanical preparation was performed using the crown-down technique with Protaper Universal Rotary NiTi files (Dentsply, Maillefer, Switzerland).  Figure 3 shows the photograph of the chamber floor after instrumentation. Final irrigation was done with 20 mililiter 2.5% sodium hypochlorite followed by 20 milliliter normal saline irrigation. The canals were dried with paper points and obturated using cold lateral compaction of gutta-percha (GAPADENT Co. LTD, China) and AH 26 sealer (Dentsply Tulsa). The access was temporarily restored with Cavit (ESPE, Seefeld, Japan). A final radiograph (Figure 4) was taken and the patient was referred to take a cone-beam computed tomography (CBCT) scan for approving this rare and unusual root canal anatomy of the tooth and other possible anomalies.

**Figure 5:**
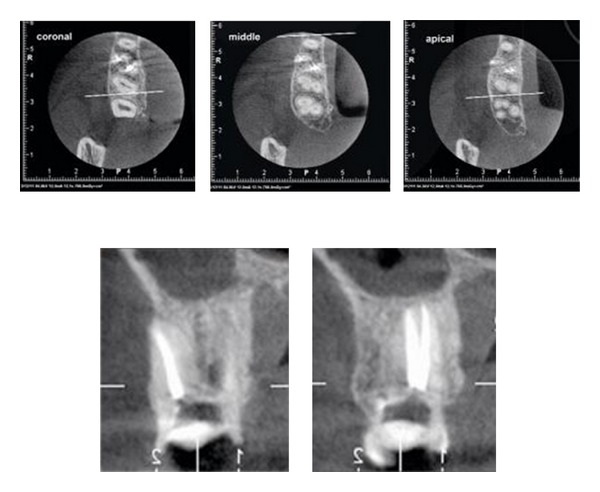
CBCT evaluation.

CBCT revealed the presence of 2 roots including one palatal root with 2 canals; each with a distinct root canal having separate orifices and separate exits and a large buccal root canal (Figure 5). The CBCT data confirmed our clinical and radiographic understanding of canal configuration.

**Figure 6:**
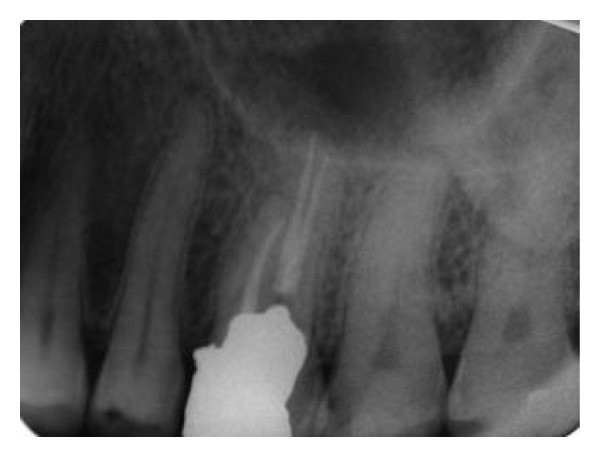
12 months follow up.

Patient was recalled 12 months later. The tooth was clinically and radiographically asymptomatic (Figure 6).

## 3. Discussion 

The present paper describes the nonsurgical endodontic management of an unusual maxillary first molar with two roots. This case is considerable because of having two palatal canals in a palatal root and one buccal canal in a buccal root. Cleghorn et al. (2006) conducted a comprehensive review of the root and root canal morphology of the maxillary first molar [2]. A thorough literature search in PubMed site was done by the author revealed that the present case is apparently the first reported case of endodontic management of a maxillary first molar with two roots (two palatal canals in one root and a single buccal root canal) that was reported.

The 2-rooted type of the maxillary first molar is rarely reported. Its incidence in the literature is 3.9% [2].The fusion of the two buccal roots has the prevalence of 0.4% in maxillary first molars [9]. Fava (2001) reported a case of maxillary first molar with two roots; two canals in the buccal root (Vertucci type IV) and one palatal root canal [9]. Nevertheless, presence of only one buccal root with one canal is extremely rare [3, 9]. Gopikrishna et al. (2008) presented the endodontic management of a maxillary first molar with a single-fused buccal root with two canals and 2 palatal roots [3]. After confirmation of this unusual morphology by spiral computerized tomography, incidentally they mentioned that the other maxillary first molar in their reported patient had also a similar morphology, but it had only one buccal canal in its single buccal root. 

Incidence of two palatal canals in maxillary first molars is also low (1%). There are some case-reports have been described two palatal canals in one palatal root in maxillary first molars as presented 4 in Table 1. Holderrieth and Gernhardt [7] and Aggarwal et al. [8] reported cases having two palatal canals in a single palatal root representing Vertucci type IV was wrong canal configuration as seen in the presented case. 

Radiographs produce only a 2-dimensional image, resulting in superimposition and distortion. Since, it cannot be useful in cases with complex root canal anatomy, it is crucial to use all of the armamentaria to diagnose and treat the entire root canal system [10]. According to previous studies [1, 2, 4, 8] where computerized tomography (CT) was used for definitive diagnosis of morphologic abnormalities in the root canal anatomy, CBCT of the involved tooth was conducted in the present case.

## 4. Conclusion

The present case was the endodontic management of a maxillary first molar with two palatal canals in one root and one buccal root canal. Although the incidence of a second palatal canal and even a single buccal root canal is not high, it is important to take these abnormalities into consideration during root canal therapy of maxillary molars in order to prevent accidents and ensure successful long-term outcome. We also accentuate the role of CBCT as an objective analytical tool to ascertain root canal morphology in unusual cases.

## Figures and Tables

**Table 1:** Review of case reports of two palatal canals in one palatal root in maxillary first molar.

Investigators	Study type	Key information
Johal 2001 [6]	Clinical RCT	2MB, 1DB, 2P
Holderrieth and Gernhardt 2009 [7]	Clinical RCT	2 cases: 2MB, 1DB, 2P
Aggarwalet al. 2009 [8]	Spiral CT	1MB, 1DB, 2P
Deepalakshmi et al. 2009 [1]	Spiral CT	2MB, 1DB, 2P

MB: mesiobuccal, DB: distobuccal, P: palatal.
